# Post-operative C-reactive protein and white blood cells changes pattern following spinal deformity surgery and its clinical correlation

**DOI:** 10.1186/s13018-023-04288-1

**Published:** 2023-10-24

**Authors:** Yehia Elbromboly, Mohamed Abdallah Esawy

**Affiliations:** Faculty of Medicine, Zagazig University, Zagazig, Egypt

## Abstract

**Objective:**

Following the changing pattern of post-operative CRP and WBC counts and compare them to the patient’s clinical condition to find which one is better for early detection of early infection.

**Methods:**

A total of 50 patients who underwent spinal deformity correction surgery without clinical signs of infection such as fever, wound redness, or discharge were enrolled in this prospective study. The C-reactive protein (CRP) and white blood cell (WBC) counts were measured the 2nd and 7th post-operative days. We try to detect the type of correlation between both CRP and WBCs level and clinical condition of patient regarding wound local condition.

**Results:**

All cases showed high CRP by the 2nd day post-operative which decreased significantly but not reaching normal levels even by the 7th day. All cases showed elevated WBCs count by the 2nd day which decreased to normal levels by the 3rd day in 86% of patients, and by the 7th day, 94% of cases showed normal levels. In addition, WBCs in the 2nd day post-operative significantly positive correlated with fusion level and operative time. There was no significant correlation between WBCs and blood transfusion or age. No significant correlation between CRP and number of fusion levels, blood transfusion nor operative time.

**Conclusion:**

WBCs count returned earlier to normal levels than CRP in our cases, so monitoring early changes in the 1st week in WBCs count pattern is more indicative of an ongoing infectious process.

## Study design

Prospective study.

## Introduction

Post-operative infection is a fearful complication that faces any spine deformity surgeon. The incidence of surgical site infection (SSI) following spinal surgery differs significantly according to the type of surgery, duration, and its complexity. Multiple factors may pose increased risk of developing SSI including diabetes, revision surgery, high body mass index, and associated syndromes as neurofibromatosis [1].

Patients with post-operative infections usually develop specific symptoms such as swelling, skin inflammation, exaggerated pain, and occasionally fever. Additionally, any inflammatory process is associated with a surge in the production of the acute-phase reactants, namely, C-reactive protein (CRP) increasing its serum levels [2]. The CRP was first described by Tillett and Francis in pneumonia [3] and with time and continuous research has been established as a reliable parameter in detecting and monitoring different types of infections [4].

White blood cells (WBC) count is not consistently elevated in infection, especially in chronic cases. Thus, its poses little importance in following up infection. On the other hand, its often elevated in acute infections [5].

Medical imaging does not attain a high positive predictive value in the evaluation of post-operative infection. Consequently, laboratory investigations are more valuable, especially, CRP and WBC, as they have been studied extensively [1].

The purpose of this study is to determine the normal changing pattern of CRP and WBC in the post-operative period in spinal deformity corrective surgery in the absence of infection to serve as a benchmark in detecting early post-operative SSI. Additionally, evaluating the effect of different variables such as blood transfusion, length of surgery, and fusion levels on the pattern of change in the CRP and WBC to help differentiate between normal pattern and SSI.

## Patients and methods

This is a prospective study done at a single spine center. The first 50 patients who were indicated for spinal deformity surgery and fit our inclusion criteria were included in the study after obtaining informed consent.

The inclusion criteria were patients who had a spinal deformity (adolescent idiopathic scoliosis, neuromuscular scoliosis, congenital scoliosis, and Sherman kyphosis) needing surgical correction and otherwise health individuals.

The exclusion criteria were presence of an autoimmune disease, chronic infection, cancer, use of immunosuppressive drugs, chronic illnesses, or history of surgery in the past 3 months.

Our aim was to illustrate the normal pattern of WBC and CRP changes in the 2nd and 7th post-operative days and to detect their relations with blood transfusion, age, fusion levels, and operative time.

All patients were administered—as per hospital protocol—Cefepime (after a sensitivity test) half an hour before surgical incision which was repeated every 4-h intra-operative in lengthy procedures followed by two consecutive doses every 12 h post-operatively.

The data were collected through history taking, clinical examination, and laboratory investigation. The data were entered into Microsoft Excel software then imported into Statistical Package for the Social Sciences (SPSS version 20.0) software for analysis. The following tests were used to test differences for significance. Differences between quantitative paired by paired t, correlation by Pearson's correlation or Spearman’s. *P* value was set at < 0.05 for significant results and < 0.001 for high significant result.

## Results

Fifty patients were included in our study. The mean age of our study group was 16 years (range 5–36), sex distribution was 33 females and 17 males. All patients did not have any clinical, radiological nor laboratory signs of infection up to 1-year follow-up.

The distribution of diagnosis is outlined in Table 1. The mean operative time was 4 h 45 min (range 3–10). The mean number of fusion levels was 13 (range 7–16). As for blood transfusion, 26 cases received intra-operative blood transfusion, and 23 cases received post-operative blood transfusion.

**Table 1:** Diagnosis distribution

Diagnosis	
Adult scoliosis	4
Adolescent idiopathic scoliosis	18
Congenital scoliosis	7
Idiopathic early-onset scoliosis	7
Marfan syndrome	2
Neurofibromatosis	3
Neuromuscular scoliosis	2
Syndromic early-onset scoliosis	1
Sherman kyphosis	6
Total	50

### Overall changes in WBCs count and CRP levels

WBCs count and CRP levels were measured according to this routine: WBCs at the 1st, 2nd, 3rd, and 7th day post-operative while CRP levels at the 2nd, and 7th day post-operative.

Our findings demonstrated that by the 3rd day post-operative, 86% of cases showed normal WBCs count while by the 7th day post-operative, 94% of cases showed normal WBCs count. All cases showed high CRP by the 2nd day which decreased significantly but not reaching normal levels even by the 7th day post-operative.

### Changes in WBCs and CRP levels in relation to different variables

WBCs in the 2nd post-operative day showed significant positive correlation with the number of fusion levels. Additionally, there was a positive correlation between the operative time and WBCs in the 7th post-operative day. There was no significant correlation between WBCs, blood transfusion, and age of the patients. No correlation was detected between CRP, number of fusion levels, blood transfusion, or operative time.

## Discussion

Post-operative infection is a feared complication especially in spine deformity surgery; hence, early detection is of paramount importance. Post-operative infections could manifest itself with different symptoms including local pain, swelling, wound inflammation, and general symptoms as fever. Nevertheless, all these symptoms cannot accurately diagnose infection [1].

Usually, the catastrophic picture of acute infection, e.g., wound dehiscence, exposed implants, and septicemia, does not appear rapidly. On the contrary, there is a gradual increase in severity of symptoms and signs [6].

CRP and WBC are the most common laboratory parameters used in detecting and following up infections as they are more sensitive compared to different radiological modalities. Inflammatory process increases the production of a group of proteins known as acute-phase reactants such as CRP [2]. The CRP level is known to double in approximately 8 h in response to different stimuli [7].

To be able to use a specific laboratory marker in detecting early post-operative infection, there is a need to know its normal pattern of change in the physiological post-operative period as the operative stress normally raises them above normal. Moreover, that would clear out the confusion due to misinterpretation of normal changes in marker levels during the post-operative period as those changes are related to an unspecific tissue reaction [8]. Kang and coauthors [9] stated that CRP reaches its peak around the 3rd post-operative day and returns to normal level by the 9th day. Also, based on their findings, the WBCs reached their peak around the 2nd day and returned to normal levels around the 4th day in both primary and revision spinal fusion surgeries [9].

Another study by Khan and colleagues [10] reported that CRP levels reach their maximum in the 2nd post-operative day (POD) which is followed by a rapid decline around the 7th post-operative day. Additionally, Choi and coauthors [11] reported on a series of 20 spinal fusion surgeries that WBCs count elevated rapidly on POD 1 and the normalized after POD 3. In their study, Kraft and coworkers [12] outlined that if the expected decline in CPR levels at the POD 7 is interrupted, then a possible infection should be anticipated as they reported that WBCs levels were normalized around POD 2 or 3 in both spinal fusion and simple discectomy surgeries. On the other hand, CRP levels returned to normal around POD 14 in both surgeries.

This study aimed to determine the changing pattern of post-operative CRP and WBC counts in the spinal deformity surgeries and investigate the effects of different variables such as blood transfusions, age, fusion levels, and operative time on the CRP and WBC counts.

**Fig. 1:**
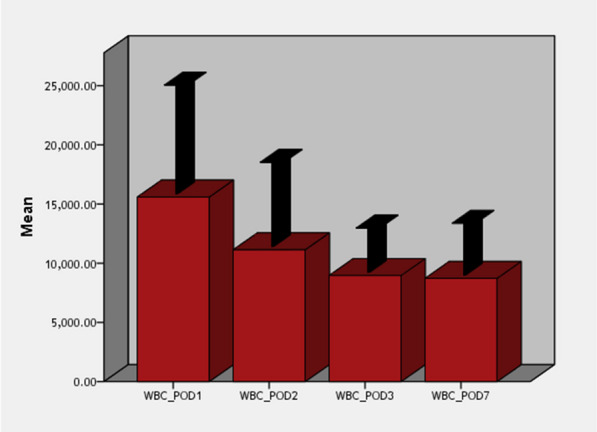
WBCs count change at post-operative days 1, 2, 3, and 7

Our results showed that by POD 3, 86% of cases showed normal WBCs count while by POD 7, 94% of cases showed normal WBCs count (Fig. 1). All cases showed high CRP by the 2nd day which decreased significantly but not reaching normal levels even by the 7th day post-operative.

**Fig. 2:**
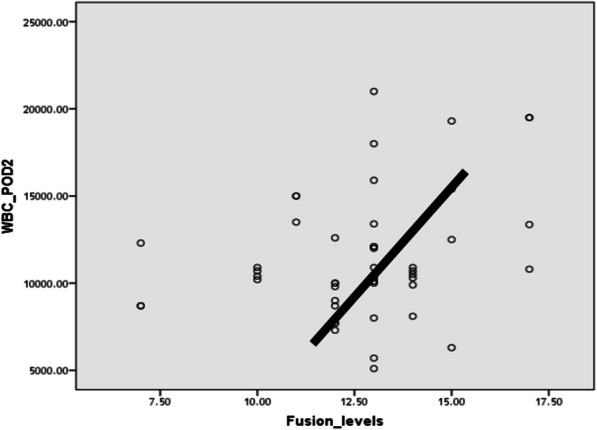
Correlation between WBCs count at the 2nd post-operative day and number of fusion levels

WBCs count in POD 2 showed significant positive correlation with fusion levels (Fig. 2), and there was also a positive correlation between operative time and WBCs at POD 7. However, there was no correlation between WBCs and blood transfusion or age. Moreover, there was no significant correlation between CRP and the above-mentioned variables.

Deirmengian and coauthors studied the rise of WBC count after arthroplasty in the lower limb, and they found similar findings [13]. Their data showed that the post-operative leukocytosis was present as a normal physiological response to surgery until the POD 4.

On the other hand, Gerven and coworkers stated that in cases of proven spondylodiscitis, the changes in the CRP level are more predictable than the changes in the serial WBC count [14] but their main focus was evaluating the treatment modality rather than diagnosing the infection itself.

Additionally, several studies have documented the sensitivity of serial CRP levels in monitoring SSI following fracture fixation [4, 15, 16] as well as in lumbar spine surgery [17] but none of them have correlated the CRP and WBCs count in patients without infections.

Apart from the different limitations of any retrospective study, the main limitations were the small sample group, heterogenous patients underlying pathology, and wide age range. However, we believe that it serves as an additional piece of information that could help the early detection of a catastrophic complication as SSI in spinal deformity surgery.

## Conclusion

In spinal deformity surgery, WBCs count return to normal levels earlier than CRP. So, monitoring changes in WBCs pattern is more reliable than CRP in the early post-operative period as an indicator for possible infectious process.

## Data Availability

All data used in this manuscript are available with the corresponding author and can be presented upon request.

## Abbreviations

SSI: Surgical site infection
CRP: C-reactive protein
WBC: White blood cells
POD: Post-operative day
